# Genome-Wide CRISPR Screens Reveal ZATT as a Synthetic Lethal Target of TOP2-Poison Etoposide That Can Act in a TDP2-Independent Pathway

**DOI:** 10.3390/ijms24076545

**Published:** 2023-03-31

**Authors:** Jeong-Min Park, Huimin Zhang, Litong Nie, Chao Wang, Min Huang, Xu Feng, Mengfan Tang, Zhen Chen, Yun Xiong, Namsoo Lee, Siting Li, Ling Yin, Traver Hart, Junjie Chen

**Affiliations:** 1 Department of Stem Cell Transplantation Research, The University of Texas MD Anderson Cancer Center, Houston, TX 77030, USA; jpark12@mdanderson.org (J.-M.P.);; 2 Department of Experimental Radiation Oncology, The University of Texas MD Anderson Cancer Center, Houston, TX 77030, USA; hzhang21@mdanderson.org (H.Z.);; 3 Department of Bioinformatics and Computational Biology, The University of Texas MD Anderson Cancer Center, Houston, TX 77030, USA

**Keywords:** ZATT, TOP2, etoposide sensitivity, CRISPR screens

## Abstract

Etoposide (ETO) is an anticancer drug that targets topoisomerase II (TOP2). It stabilizes a normally transient TOP2–DNA covalent complex (TOP2cc), thus leading to DNA double-strand breaks (DSBs). Tyrosyl-DNA phosphodiesterases two (TDP2) is directly involved in the repair of TOP2cc by removing phosphotyrosyl peptides from 5′-termini of DSBs. Recent studies suggest that additional factors are required for TOP2cc repair, which include the proteasome and the zinc finger protein associated with TDP2 and TOP2, named ZATT. ZATT may alter the conformation of TOP2cc in a way that renders the accessibility of TDP2 for TOP2cc removal. In this study, our genome-wide clustered regularly interspaced short palindromic repeats (CRISPR) screens revealed that ZATT also has a TDP2-independent role in promoting cell survival following ETO treatment. ZATT KO cells showed relatively higher ETO sensitivity than TDP2-KO cells, and ZATT/TDP2 DKO cells displayed additive hypersensitivity to ETO treatment. The study using a series of deletion mutants of ZATT determined that the N-terminal 1–168 residues of ZATT are required for interaction with TOP2 and this interaction is critical to ETO sensitivity. Moreover, depletion of ZATT resulted in accelerated TOP2 degradation after ETO or cycloheximide (CHX) treatment, suggesting that ZATT may increase TOP2 stability and likely participate in TOP2 turnover. Taken together, this study suggests that ZATT is a critical determinant that dictates responses to ETO treatment and targeting. ZATT is a promising strategy to increase ETO efficacy for cancer therapy.

## 1. Introduction

Topoisomerases resolve topological stresses from DNA during transcription and replication through transient cleavage of DNA strands [1]. During cleavage reaction, the tyrosine in the catalytic active site of topoisomerases is covalently linked to the phosphate backbone of DNA, which generates the covalent DNA–protein complexes (DPCs) [2]. The formation of DPCs is the basis of the anticancer effect for chemotherapeutic drugs such as camptothecin (CPT) targeting type I topoisomerases (TOP1) and etoposide (ETO) targeting type II topoisomerases [3].

TOP2 forms a dimer and cleaves both strands of a DNA duplex, which results in the formation of the TOP2 cleavage complex (TOP2cc) [4]. TOP2cc by itself is not toxic, since TOP2cc formation is usually transient and DNA breaks are buried inside the TOP2 dimer. Moreover, the DNA ends in TOP2cc are also protected by their covalently-linked TOP2 protein moiety. Therefore, these DNA ends are not detected by DNA damage signaling pathways, nor are they substrates for any DNA double-strand break (DSB) repair mechanisms including nonhomologous end joining (NHEJ) and homologous recombination (HR), since all these events require the presence of free DNA ends. However, the collision of TOP2cc with the ongoing transcription or replication fork would lead to the exposure of TOP2cc, which is subsequently degraded by the proteasome and other proteolytic pathways [5]. The proteolytic processing of TOP2cc results in the exposure of DNA double-stranded breaks, which now become irreversible and highly toxic [6]. Thus, TOP2cc processing is tightly regulated to avoid this irreversible step and to prevent the generation of genotoxic lesions such as DSBs.

Tyrosyl-DNA phosphodiesterases 2 (TDP2) is the only enzyme known to be specifically involved in the repair of TOP2cc [7]. Since the TOP2-induced DSBs are derived from that the enzyme remains covalently linked to the 5′ end of DNA through a phosphotyrosyl bond, and TDP2 restores 5′-phosphate termini of DSB in preparation for DNA ligation [8]. TDP2 by itself could not cleave intact TOP2cc in vitro, suggesting that proteolytic degradation of TOP2cc is needed before TDP2 can remove the remaining TOP2cc [9]. More recently, the zinc finger protein associated with TDP2 and TOP2, named ZATT, has also been shown to be required for the removal of TOP2cc by TDP2 [10]. 

ZATT (previously known as zinc finger protein 451; ZNF451) was initially identified as a promyelocytic leukemia bodies (PML)-associated protein, which has small ubiquitin like modifier two (SUMO2) and SUMO3 (SUMO2/3)-specific E3 ligase activity [11]. It was also identified as the SUMO ligase that can promote TOP2cc SUMOylation and its direct removal by TDP2 [12]. ZATT catalyzes SUMO2/3 conjugation to TOP2cc which alters the conformation of TOP2cc and promotes TDP2 recruitment [13]. This E3 SUMO ligase function is restricted to the very N-terminal 56 residues of ZATT, which contains the tandem SUMO-interacting motifs (SIMs). The potential functional significance of the rest of ZATT, which comprises ~1000 residues and contains the central 11 zinc fingers and the C-terminal ubiquitin-interacting motif (UIM), has not been studied yet. More recently, ZATT has also been shown to act with TOP2 and Plk1-interacting checkpoint helicase (PICH) in replication fork reversal [14]. Although ZATT is now emerging as another regulator of TOP2, how ZATT participates in the processing of TOP2 poison–induced DNA damage has not yet been fully elucidated.

In this study, ZATT KO cells showed relatively higher ETO sensitivity than TDP2 KO cells. Furthermore, ZATT/TDP2 double knockout (DKO) cells displayed an additional increase in sensitivity to ETO treatment. These data suggest that ZATT has a TDP2-independent role in ETO-induced TOP2cc repair. Additionally, we determined the regions of ZATT involved in its interaction with TOP2 and ETO sensitivity using a series of internal deletion mutants of ZATT. Moreover, the depletion of ZATT led to accelerated TOP2 degradation after ETO treatment and a shortened half-life of the TOP2 protein following cycloheximide (CHX) treatment. These results indicate that ZATT may increase TOP2 stability and contribute to TOP2 turnover. Our clustered regularly interspaced short palindromic repeats (CRISPR) screens also revealed that several genes whose loss leads to increased ETO sensitivity upon ZATT loss are related to cell-cycle regulation and/or cell-cycle checkpoint control. Together our results uncover a TDP2-independent function of ZATT, which may help in the development of therapeutic strategies that improve ETO efficacy.

## 2. Results

### 2.1. ZATT KO Cells Showed More Enhanced ETO Sensitivity Than TDP2 KO Cells

**Figure 1:**
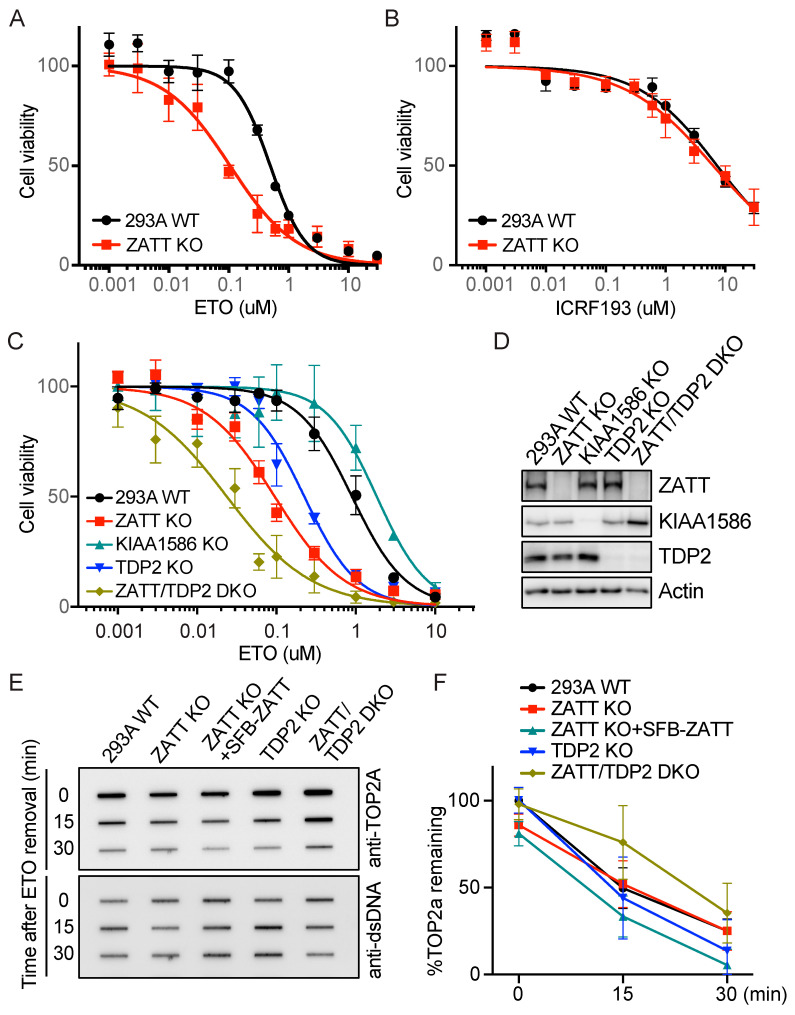
ZATT KO cells showed higher ETO sensitivity than TDP2 KO cells. (**A**,**B**) Cell survival was measured using a CellTiter-Glo assay in HEK293A WT and ZATT KO cells treated with indicated drugs for 3 days. Each experiment was repeated three independent times done in triplicate each time. Data were represented as mean ± SD. (**C**) Cell survival was measured by a CellTiter-Glo assay after 3 days in the presence of the indicated concentrations of ETO. Each experiment was repeated three independent times done in triplicate each time. Data were represented as mean ± SD. (**D**) HEK293A were infected with pLenti-V2-sgRNAs that target ZATT, KIAA1586, or TDP2 to generate KO cells. Whole-cell extracts were prepared and subjected to western blotting with the indicated antibodies. (**E**) Cells were pretreated with 20 μM MG132 for 1.5 h and then incubated with 100 µM ETO for 1 h. Cells were incubated with fresh medium containing MG132 for indicated recovery times and subjected to RADAR assay. The removal rate of TOP2cc was analyzed using immune-slot blotting with TOP2 antibody. dsDNA antibody was used to monitor the amount of DNA loaded in each sample. (**F**) Quantitation of TOP2cc from three independent experiments as shown in (**E**). TOP2cc were measured by densitometric analyses of slot blot signals and plotted as averages and standard deviations.

It has been reported that the knockdown of ZATT increases cellular sensitivity to ETO but not to other DNA-damaging agents [12]. To confirm that ZATT is specifically involved in the TOP2 poison-induced DNA damage response, we generated the ZATT knockout (KO) cells by CRISPR/Cas9 editing and checked the cell viability in response to various DNA damaging agents. Consistent with previous studies, ZATT KO cells showed reduced cell survival when compared to wild-type (WT) cells in response to ETO, but not to other DNA damaging agents including CPT, mitomycin C (MMC), cisplatin, hydroxyurea (HU), or ionizing radiation (IR) (Figure 1A and Figure S1A). Additionally, we checked the cell viability with ICRF 193 treatment which is a catalytic inhibitor of TOP2. We showed that ZATT KO cells were not sensitive to ICRF 193 (Figure 1B), indicating a specific function of ZATT in response to TOP2 poison ETO.

Previous studies revealed that ZATT N-terminal SIMs are important for the SUMO E3 ligase activity which facilitates the SUMOylation of TOP2cc and its removal by TDP2 [12,15]. ZATT and E3 SUMO-protein ligase KIAA1586 (KIAA1586) are neighboring genes on chromosome 6p12.1 in human cells, and KIAA1586 protein shares extensive homology with ZATT at its N-terminal SIMs but has very different C-terminal sequences. Since KIAA1586 also has E3 SUMO ligase activity [16], we investigated whether KIAA1586 is involved in ETO-induced TOP2cc or other DNA damage repair. We carefully designed sgRNA for KIAA1586 due to its sequence homology with ZATT and confirmed the knockout by TA cloning and sequencing to avoid any off-target effect. KIAA1586 KO cells did not show any increased sensitivity to DNA-damaging agents, including ETO (Figure 1C and Figure S1B), suggesting that KIAA1586 is not required for TOP2cc repair. 

We then examined the ETO sensitivity in TDP2 KO cells. TDP2 KO cells showed increased ETO sensitivity, though, surprisingly, was relatively milder when compared to ZATT KO cells (Figure 1C). Furthermore, ZATT/TDP2 double knockout (DKO) cells, which were confirmed by immunoblotting (Figure 1D), displayed an additive effect in response to ETO treatment, suggesting that ZATT has a TDP2-independent role in ETO-induced TOP2cc repair. We also checked the time course of TOP2cc removal using rapid approach to DNA adduct recovery (RADAR) assays with antibodies recognizing TOP2A. To investigate the proteasome-independent manner [17], we pretreated cells with proteasome inhibitor MG132 and maintained the presence of MG132 until indicated recovery times. We found that the TOP2cc removal rates were unaffected by ZATT or TDP2 single KO in the presence of MG132, however, only ZATT/TDP2 DKO cells showed delayed removal of TOP2cc (Figure 1E,F). These data suggest that ZATT has a TDP2-independent function, which can act as an alternative pathway for TOP2cc processing.

### 2.2. ZATT Isoform 3 Was Not Able to Rescue Etoposide Sensitivity in ZATT KO Cells

**Figure 2:**
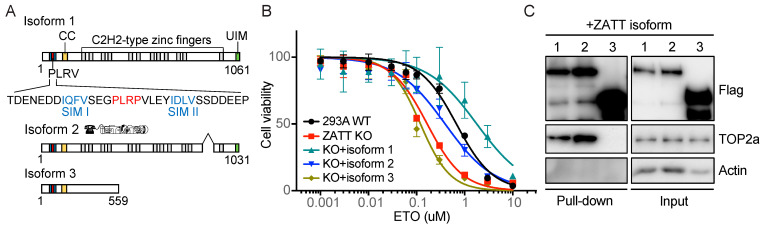
ZATT isoform 3 was not able to rescue ETO sensitivity in ZATT KO probably due to a lack of interaction with TOP2 (**A**) Schematic diagrams of ZNF45 isoforms. ZATT isoform 1 consists of 2 N-terminal SUMO interacting motifs (SIMs), 11 zinc finger domains, and a ubiquitin interacting motif (UIM). (**B**) ZATT KO cells were transfected with constructs encoding SFB-ZATT isoform 1, isoform 2, and isoform 3. Cell survival was measured by a CellTiter-Glo assay after 3 days in the presence of the indicated concentrations of ETO. Each experiment was repeated three independent times done in triplicate each time. (**C**) Cells were transfected with constructs encoding SFB-ZATT isoforms and subjected to pull-down with streptavidin. Western blotting was conducted with antibodies as indicated. The input lanes contain 2% of input protein.

Human ZATT has three isoforms, which all contain the N-terminal tandem SIMs (Figure 2A). Isoform one is the longest, while isoform two lacks residues 870–917 and isoform three has a distinct C-terminal LAP2 domain. We reconstituted ZATT KO cells with ZATT isoforms and showed that isoforms one and two were able to rescue ETO sensitivity in these cells (Figure 2B). We showed that ZATT isoform three, which contains the identical N-terminal 56 residues, though a different C-terminal region, was not able to rescue ETO sensitivity in ZATT KO cells, indicating that in addition to its N-terminal SIMs, other regions of ZATT also contribute to its functions in ETO sensitivity. Our proteomics analysis of TOP2 and other topoisomerases uncovered ZATT as the major and specific TOP2-associated protein. Moreover, the interaction of ZATT with TOP2A or TOP2B did not change significantly in response to DNA damage or ETO treatment, indicating that ZATT is a part of the TOP2A–2B complex. We further analyzed the ZATT isoforms binding properties for endogenous TOP2 using immunoprecipitation (IP) experiments. ZATT isoforms one and two interact with TOP2A (Figure 2C), though isoform three did not. These data suggest that isoform three may not be able to rescue ETO sensitivity in ZATT KO cells due to the lack of TOP2 interaction. 

### 2.3. The N-Terminal 1–168 Residues of ZATT Is Essential for Its Interaction with TOP2

**Figure 3:**
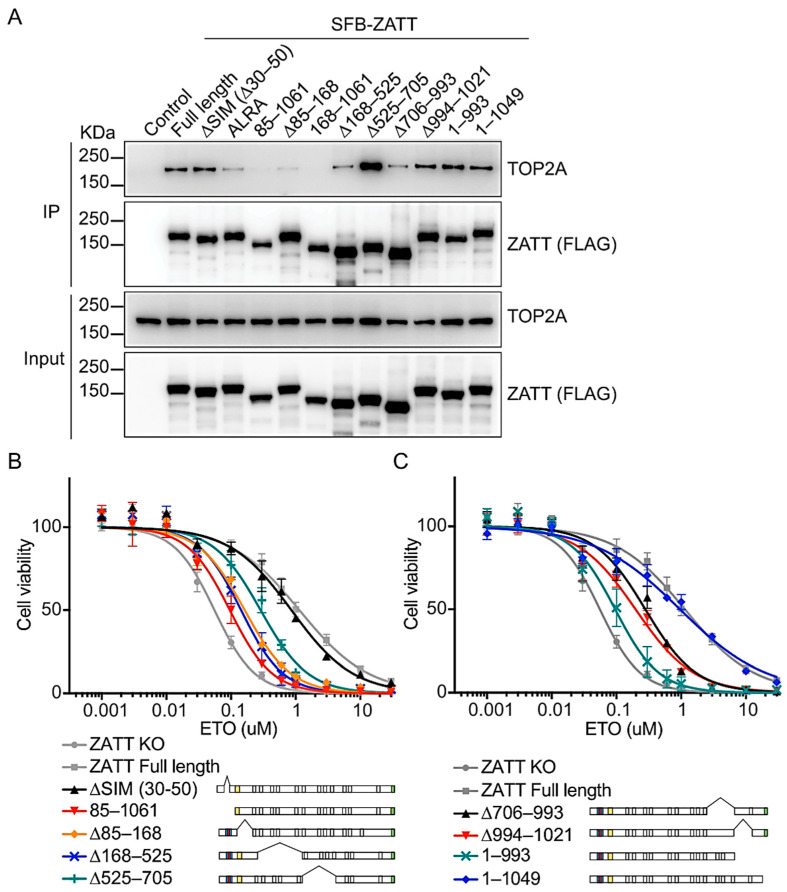
The N-terminal 1–168 region of ZATT is essential for its interaction with TOP2 and both the N- and C-terminal regions of ZATT affect etoposide sensitivity. (**A**) HEK293A cells were transfected with constructs encoding truncated SFB-ZATT or internal deletion mutants and subjected to pull down with streptavidin. Western blotting was conducted with antibodies as indicated. (**B**,**C**) ZATT KO cells were transfected with indicated constructs and selected with puromycin. Cell survival was measured by a CellTiter-Glo assay after 3 days in the presence of the indicated concentrations of ETO. Each experiment was repeated three independent times done in triplicate each time. Data were represented as mean ± SD. The legends below are schematic depictions of ZATT deletion mutants used in this study.

The previous structural study has shown that the two SIMs of ZATT are separated by an intervening PLRP motif which is important for SUMO E3 ligase activity [18]. Therefore, we introduced alanine substitutions of Pro38 and Pro41 (PLRP→ALRA) and deletion of 20 amino acids for N-terminal tandem SIMs (ΔSIM; Δ30–50). The ΔSIM mutant preserved a WT-like, strong interaction with TOP2A, while the ALRA mutant showed decreased TOP2A binding (Figure 3A, lanes 3–4). Deletions were then further extended to the entire N-terminal regions (i.e., 85–1061 and 168–1061) or after the SIMs (i.e., Δ85–168 and Δ168–525). Interestingly, interaction with TOP2 was abolished or significantly reduced for the N-terminal deletion mutant 85–1061 and a larger deletion mutant 168–1061 as well (Figure 3A, lanes 5 and 7). Deletion of residues 85–168 led to reduced TOP2A binding, though not as much as other N-terminal deletion mutations. The Δ168–525 and Δ706–993 also showed weakened interaction with TOP2, which might be due to the large deletion of protein regions. Thus, the TOP2 interaction region of ZATT was mapped to amino-acids 1–168.

Next, we analyzed the ETO sensitivity using these ZATT deletion mutants. Although the only known function of ZATT is its SUMO E3 ligase activity which requires N-terminal SIMs [15], the ΔSIM mutant showed WT-like ETO sensitivity without detectable differences in the cell survival assay (Figure 3B). Meanwhile, the N-terminal deletion mutants were not able to rescue ETO sensitivity in the ZATT KO cells, which is consistent with the loss of TOP2 interaction. Interestingly, the C-terminal deletion mutant 1–993 also could not rescue the increased ETO sensitivity in ZATT KO cells (Figure 3C), even though it was able to interact with TOP2 (Figure 3A, lane 12). These data indicate that in addition to the interaction with TOP2 through the N-terminal of ZATT, the C-terminal regions of ZATT also contribute to its functions in ETO sensitivity.

### 2.4. ZATT Depletion Leads to Destabilization of TOP2 Protein 

**Figure 4:**
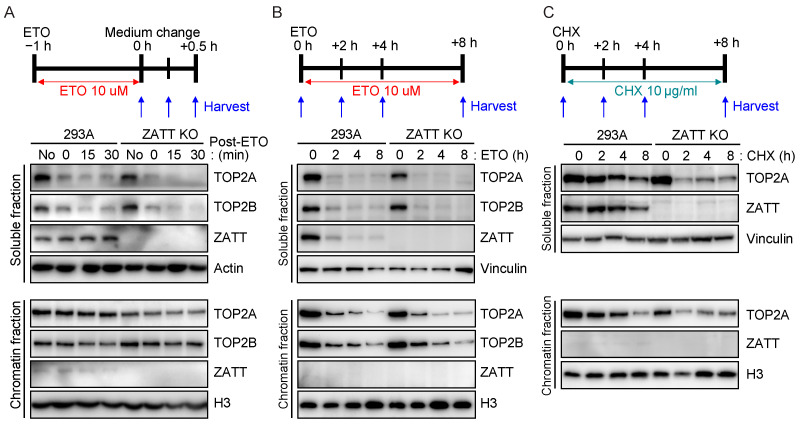
Depletion of ZATT led to the destabilization of TOP2A protein. (**A**) Cells were treated with 10 μM ETO for 1 h and changed to fresh medium. Cells were harvested at an indicated time and separated into soluble and chromatin fractions. The TOP2 protein level was analyzed by Western blotting with the indicated antibodies. (**B**) Cells were treated with 10 μM ETO for an indicated time. Soluble and chromatin fractions were prepared and subjected to Western blotting with the indicated antibodies. (**C**) Cells were treated with 10 μg/mL CHX for an indicated time and separated into soluble and chromatin fractions. The TOP2 protein level was analyzed by Western blotting with the indicated antibodies.

The ZATT C-terminal region contains UIM, which is a short peptide motif of about 20 residues and was known to recognize ubiquitin. Since many UIM-containing proteins can participate in ubiquitylation [19], we assume that ZATT may also have functions in ubiquitin metabolism. It was reported that the ubiquitin–proteasome system is primarily responsible for regulating the stability and activity of TOP2 [20]. TOP2 is ubiquitinated and degraded by proteasome upon ETO treatment and this process may occur prior to TOPcc removal by TDP2 [10]. We found that ZATT depletion led to accelerated TOP2A degradation in a soluble fraction after ETO treatment (Figure 4A). This proteolysis of TOP2A was suppressed by proteasome inhibitor MG132 both in the WT and ZATT KO cells (Figure S2). When we treated ETO for a longer period, ZATT depletion did not lead to any significant change in TOP2A degradation in soluble or chromatin fractions (Figure 4B), since TOP2A/2B has already been degraded under these conditions. Moreover, even when ETO was not present, the TOP2A protein level appeared slightly lower in ZATT KO cells compared to WT cells (Figure 4A, lane 5 and Figure 4B, lane 5). We further investigated the potential influences of ZATT on TOP2 protein stability by using the protein synthesis inhibitor cycloheximide (CHX). Treatment of CHX led to a rapid loss of TOP2A protein in ZATT KO cells (Figure 4C). Together, these data suggest that ZATT facilitates TOP2A stability and likely participates in TOP2A turnover. 

### 2.5. Genome-Wide CRISPR Screens Identified ZATT as a Critical Gene for ETO Sensitivity

**Figure 5:**
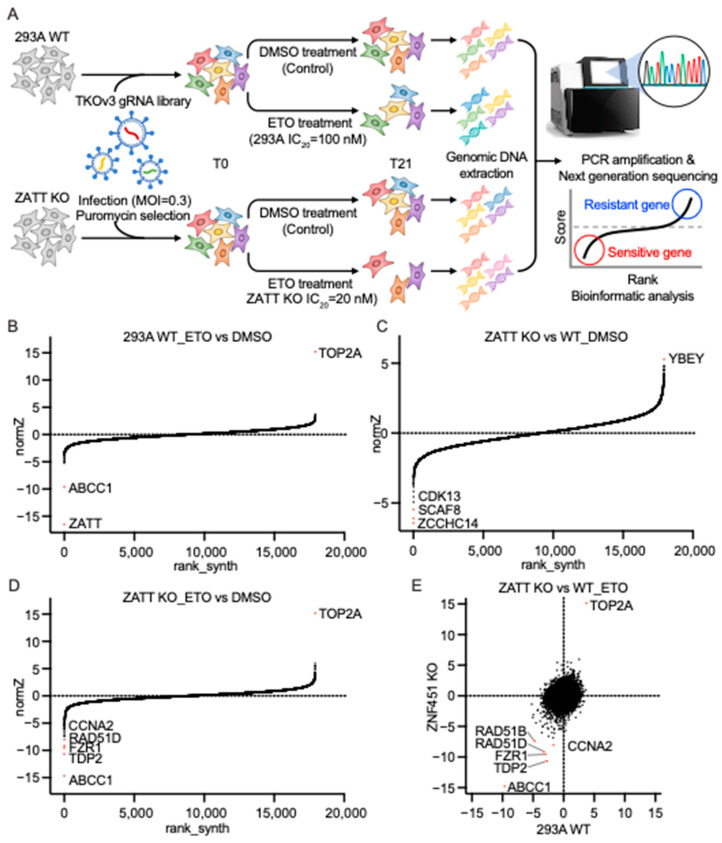
Genome-wide CRISPR screens identified ZATT as a critical gene important for ETO sensitivity. (**A**) Schematic diagram of the workflow for the CRISPR screens performed in parental 293A and ZATT KO cells with or without ETO treatment. TKOv3, Toronto Knock Out Library v3; MOI, the multiplicity of infection; DMSO, dimethyl sulfoxide; NGS, next-generation sequencing. (**B**) Ranking of ETO coessential genes based on a drug Z analysis of the results of CRISPR/Cas9 screening in 293A-WT cells. The z-score was used to define a possible synthetic lethal interaction with ETO. The ETO-treated group and DMSO treatment group in WT cells were compared. Genes whose loss of function led to ETO sensitivity appear on the left side, with a minus Z score, and genes whose loss of function led to ETO resistance appear on the right side, with a positive Z score. Top-ranked genes on either side are marked. (**C**) Ranking of ETO coessential genes based on a drug Z analysis of the results of CRISPR/Cas9 screening in ZATT KO cells. The ETO-treated group and DMSO group in ZATT KO cells were compared. (**D**) Ranking of ZATT coessential genes based on a drug Z analysis of the results of CRISPR/Cas9 screening. The z score was used to define a possible synthetic lethal interaction with ZATT. The DMSO ZATT KO cell group and DMSO 293A-WT cell group were compared. (**E**) Combinational comparison of ETO coessential genes between 293A-WT and ZATT KO cells. The z-scores from C and D were used.

To gain further insights into the potential function of ZATT and the processes involved in TOP2cc repair, we performed genome-wide CRISPR screens in parental 293A cells and ZATT KO cells, with or without ETO treatment (Figure 5A). We used a drug Z analysis to compare the ETO-treated group with the no treatment (NT) group in WT or ZATT KO cells to determine ETO-sensitivity profiling (Tables S1–S3). The ranking of ETO coessential genes showed ATP-binding cassette transporter one (ABCC1) as the high-ranked gene that sensitizes cells to ETO treatment and TOP2A as the top gene that confer ETO resistance in both WT and ZATT KO cells, indicating that our results are highly reliable (Figure 5B,D). ABCC1, also called multidrug resistance protein-1 (MRP1), is a member of the ATP-binding cassette transporter family and a unidirectional efflux transporter for many xenobiotics, including ETO [21]. Consistent with our above data, ZATT appeared as the top-ranked sensitive gene in parental 293A cells (Figure 5B). TDP2 was a top-ranked gene that showed synthetic lethality to ETO treatment in ZATT KO cells (Figure 5D). These results clearly indicated that ZATT has TDP2-independent roles in response to the ETO and the ZATT expression level can be used as an important marker for evaluating ETO sensitivity.

In addition to ABCC1 and TDP2, fizzy and cell division cycle 20 related one (FZR1) and Cyclin-A2 (CCNA2) are candidates that sensitize both 293A cells and ZATT KO cells to ETO (Figure 5E). FZR1 regulates cell division at the end of mitosis by activating the anaphase-promoting complex (APC) and CCNA2 can control both the G1/S and the G2/M transition by binding to cyclin-dependent kinase one (CDK1) or cyclin-dependent kinase two (CDK2) [22,23]. In ZATT KO cells, the depletion of FZR1 or CCNA2 made cells more sensitive to ETO (Figure 5D), which suggests that survival pathways that function in the absence of ZATT may depend on the cell cycle progression and/or the cell cycle checkpoint signaling pathway. To obtain synthetic lethality profiling with the loss of ZATT, we compared the ZATT KO NT group with the 293A NT group (Figure 5C). ZATT showed synthetic lethality with cyclin-dependent kinase 13 (CDK13) and SR-related and CTD-associated factor eight (SCAF8) which are known to regulate RNA polymerase II transcription and mRNA processing. These data suggested that transcriptional regulation is probably important for cell survival upon ZATT depletion.

## 3. Discussion

In this study, ZATT KO cells showed ETO sensitivity, which is relatively higher than that observed in TDP2 KO cells. Furthermore, ZATT/TDP2 DKO cells displayed an additive effect in response to ETO treatment, suggesting that ZATT has a TDP2-independent role in ETO-induced TOP2cc repair. These data agree well with our unbiased CRISPR screen results, which showed that while loss of ZATT led to increased ETO sensitivity in parental 293A cells, loss of TDP2 showed additive ETO sensitivity in ZATT KO cells.

This study also determined the regions of ZATT involved in its interaction with TOP2 and ETO sensitivity. Surprisingly, deletion of the N-terminal SIMs on ZATT did not change the cell viability response to ETO, however, the larger deletion of the N-terminal region corresponding to residues 1–85 abolished its interaction with TOP2 and showed higher sensitivity to ETO. Deletion of residues 85–168 and 168–525 also leads to weakened interaction with TOP2 and increased ETO sensitivity. Interestingly, the deletion of the C-terminal residue 994–1061 showed increased sensitivity to ETO despite retaining the ability to interact with TOP2. ZATT isoform three, which contains the identical N-terminal, though different C-terminal region, was not able to rescue ETO sensitivity in ZATT KO cells, indicating that in addition to its N-terminus, other regions of ZATT also contribute to its functions in ETO sensitivity.

A previous study has shown that depletion of the ubiquitin-conjugating enzyme E2-EPF sensitized HeLa cells to ETO and increased TOP2α protein levels, suggesting that inhibiting the degradation of TOP2 may enhance TOP2 poison efficacy [17]. In contrast, our data showed that depletion of ZATT showed accelerated TOP2A degradation after ETO treatment and sensitized cells to ETO. A recent study showed that either reduction or overexpression of TOP2 causes severe growth defects in organ development [24], suggesting that TOP2 protein must be maintained at an optimal level for cell survival. Phosphatase and tensin homolog (PTEN) was described to regulate the stability of TOP2A [25]. Depletion of PTEN enhances the degradation of TOP2A, leading to the dysfunction of the decatenation checkpoint. We found that the depletion of ZATT showed a shortened half life of the TOP2A protein following CHX treatment, though ZATT loss did not sensitize cells to the TOP2 catalytic inhibitor ICRF 193. Future experiments will be needed to uncover the precise mechanisms of ZATT in regulating TOP2A protein stability and turnover. Due to the existence of TOP2A and TOP2B in human cells, and the lethality of the knockout of Top2A, it is challenging to assess the regulation of TOP2A. To simplify the functional analysis of ZATT-dependent TOP2A regulation, we would initially generate TOP2B KO cells, followed by the inducible removal of TOP2A using the degradation tag (dTAG) system with the addition of a dTAG ligand. The dTAG system, developed by Dr. Gray and colleagues, is highly effective for studying essential and relatively abundant proteins [26]. We anticipate that treatment of dTAG-TOP2A cells with dTAGv-1, which induces the degradation of the TOP2A protein, which would cause the cells to stop proliferating. By reconstituting the cells with wild-type, and various mutants, of TOP2A, we can determine whether the ZATT–TOP2A interaction or ZATT-mediated SUMOylation is required for TOP2 turnover and its functions in cell proliferation and chromosome segregation. 

Our CRISPR screens revealed that several genes whose loss led to increased ETO sensitivity with ZATT loss are related to cell-cycle regulation and cell-cycle checkpoint control. FZR1 (also known as Cdh1) is a cofactor of the APC–C complex that modulates cell proliferation by targeting multiple cell-cycle regulators for ubiquitin-dependent degradation. In FZR1-depleted mouse and human cells, both the protein level and protein stability of TOP2A were increased [27]. Our screening results further emphasized that the protein level of TOP2 is a key determinant of the cellular response to ETO treatment. Phosphorylation of TOP2a by CCNA2 with Cdk1 has been shown in zebrafish [28], which was suggested to be required for S phase entry during retinal development. How this was related to the cellular sensitivity to ETO treatment is still unclear. Additional CRISPR and FACS-based screens in ZATT/TDP2 DKO cells would be helpful in identifying genes and proteins that are important for TOP2-induced DNA damage signaling beyond ZATT and TDP2. The post-translational modifications of TOP2 have been reported to influence its catalytic activity, protein stability, and subcellular distributions [29]. It is also possible that the phosphorylation status of residues in the vicinity of the TOP2A SUMOylation site can also potentially influence its SUMO modification [30]. All these results suggest a tight and important regulation of TOP2 is important for cell proliferation. 

## 4. Materials and Methods

### 4.1. Cell Lines and CRISPR/Cas9-Mediated Gene Knockout

HEK293A cells were purchased from the American Type Culture Collection (ATCC; Manassas, VA, USA) and were cultured in Dulbecco’s Modified Eagle’s Medium (DMEM) containing 10% fetal calf serum (FCS) at 37 °C with 5% CO_2_. The cell lines tested negative for mycoplasma contamination. 

HEK293A ZATT KO, KIAA1586-KO, TDP2-KO, and ZATT/TDP2 DKO cell lines were established by transfecting pLentiCRISPRv2 (addgene; Watertown, MA, USA, 52961) vector to cells. sgRNAs targeting a specific gene were designed and ligated into BsmBΙ-digested pLentiCRISPRv2. The pLentiCRISPRvs-sgRNA were then transiently transfected into cells using polyethyleneimine. Then, 24 h after transfection, cells were selected with 2 µg/mL puromycin (Sigma Aldrich; Saint Louis, MO, USA, P8833) for 2 days, and then 50 cells were seeded into a 96-well plate for 0.5 cells/well. Ten days after seeding, single clones were selected for further verification by immunoblotting and DNA sequencing. The sequences of sgRNAs used for generating knockout cells are provided below:

- ZATT-sgRNA #1: 5′ CACCGTGTTCTTGAATACATTGATC 3′
- KIAA1586 sgRNA #1: 5′ CACCCTGGAGATAAATCACTAGGG 3′
- TDP2-sgRNA #1: 5′ CACCGTCTCCCAGTCGTTCTCGGCC 3′

### 4.2. CellTiter-Glo Assay

For the cell-survival assay, 1000 cells were seeded into 96-well plates in triplicate. After 24 h, cells were incubated with serially diluted concentrations of ETO (Fisher Scientific; Waltham, MA, USA, E1383100MG) or other chemicals for 3 days. After incubation, cells were treated with CellTiter-Glo (Promega; Madison, WI, USA, G7572) following the manufacturer’s instructions, and luminescence was recorded using a BioTek Synergy™ 2 multimode microplate reader. Each experiment was performed a minimum of three times. The chemicals used in this assay included ICRF193 (Enzo; New York, NY, USA, BML-GR332-0001), camptothecin (Calbiochem; San Diego, CA, USA, 390238-25MG), cisplatin (Selleck Chemicals; Houston, TX, USA, S1166), and mitomycin C (Sigma-Aldrich; Saint Louis, MO, USA, M4287-2MG).

### 4.3. Genome-Wide CRISPR/Cas9 Screens 

The CRISPR screens, using the Toronto Knock Out Library v3 (TKOv3) gRNA library, were conducted as described previously [31,32]. Briefly, 120 million HEK293A WT cells and 200 million ZATT KO cells were infected with the TKOv3 library lentiviruses at a low MOI (<0.3). At 24 h after infection, the medium was replaced with a fresh medium containing 2 μg/mL puromycin. Two days after selection, 20 million cells were collected for T0, and then the remaining cells were split into different groups with 3 replicates containing at least 20 million cells in each group. Cells were incubated with DMSO (control) or ETO treatment and passaged every 3 days while maintaining at least 20 million cells. At day 18 and 21 (T21), 25 million cells were collected, and genomic DNA was extracted from T0 and T21 using a QIAamp Blood Maxi Kit (Qiagen; Venlo, The Netherlands). The gRNA inserts were amplified via PCR using primers harboring Illumina TruSeq adapters with i5 and i7 barcodes, as previously reported [33]. The resulting libraries were sequenced on an Illumina HiSeq 2500 system. The sequencing results were analyzed by a model-based analysis of genome-wide CRISPR/Cas9 knockout (MAGeCK) and drug Z.

### 4.4. Plasmids and Reconstitution Experiments

The pCMV6-Entry vector containing ZATT ORF was purchased from Origene (RC215720). The CDS was amplified and cloned into the pDONR201 and pDEST-SFB vectors using the gateway system. All the deletions were made in the pDONR201-ZATT vector and transferred to pDEST-SFB. 

For the generation of reconstituted cells, constructs encoding WT and mutant ZATT were transfected into ZATT KO cells. After transfection, cells were selected with puromycin, and then diluted and seeded into 96-well plates. Clones were transferred to a 24-well plate after sufficient growth and confirmed by western blotting. 

### 4.5. Western Blot Analysis 

Cells were washed once with phosphate-buffered saline (PBS; Invitrogen, Waltham, MA, USA) and then directly lysed in an SDS sample buffer (50 mM Tris-HCl, pH 6.8, 2% SDS, 10% Glycerol, 4% β-mercaptoethanol, and 0.025% Bromophenol blue) and denatured by heating to 95 °C for 10 min. To separate soluble and chromatin fractions, cells were harvested and lysed with chilled 1× NETN buffer (50 mM Tris-HCl [pH 7.4], 100 mM NaCl, 0.4% NP-40, and 1 mM EDTA) supplemented with a protease inhibitor cocktail (Roche; Penzberg, Germany, 11836153001). After high-speed centrifugation (13,000 rpm for 10 min), the supernatant was considered a soluble fraction. The left pellet was washed twice with a 1× NETN buffer, and chromatin was digested with Turbonuclease (Accelagen; San Diego, CA, USA, N0103) in a nuclease buffer (10 mM Tris-Cl, pH 8.0, 1 mM MgCl_2_) in the presence of protease inhibitors. After 15 min of incubation at 37 °C, the lysate was spun down and the supernatant was collected as a chromatin fraction.

Samples were run on SDS-PAGE (BioRAD; Hercules, CA, USA) and transferred to a PVDF membrane (Millipore; Bedford, MA, USA). After transfer, membranes were washed in a TBST solution and blocked to 5% skim milk in TBST. Membranes were analyzed by immunoblotting with the indicated antibodies and visualized by the BioRAD Chemidoc imaging system. Antibodies used in the study are the following: ZATT (Novus Biologicals; Littleton, CO, USA, NBP2-94743-0.1 ml, Sigma-Aldrich, SAB2108741-100 UL), KIAA1586 (Novus Biologicals, NBP1-86291), TDP2 (Santa Cruz Biotechnology; Dallas, TX, USA, sc-377280), Actin (Sigma-Aldrich, A5441-100UL), TOP2A (Abcam; Cambridge, UK, ab52934), Flag (Sigma-Aldrich, F3165), Vinculin (Sigma-Aldrich, V9131), and H3 (Abcam ab1791).

### 4.6. Rapid Approach to DNA Adduct Recovery Assay

To purify TOP2cc from the cells, we used the rapid approach to DNA adduct recovery (RADAR) assay, as described previously [34]. Briefly, cells were pretreated with 20 μM MG132 (Selleck Chemicals, S2619) for 1.5 h, and then ETO was added into the medium at a final concentration of 100 μM. After 1 h of incubation, cells were washed with 1× PBS and incubated with a fresh medium containing 20 μM MG132 for the indicated recovery time. Cells were lysed by an MB buffer (6 M guanidinium isothiocyanate, 10 mM Tris-HCl [pH 6.8], 20 mM EDTA, 4% Triton X-100, 1% sarkosyl, and 1% dithiothreitol). DNA was precipitated by adding 100% ethanol, then washed three times in 70% ethanol, and solubilized in 8 mM NaOH. DNA was treated with RNAase A at 37 °C for 1 h and quantified using a NanoDrop spectrophotometer (Thermo Scientific; Waltham, MA, USA). DNA was vacuum transferred to a nitrocellulose membrane using a BioDot SF 246 microfiltration apparatus (BioRad; Hercules, CA, USA) and crosslinked by UVC irradiation. Membranes were analyzed by immunoblotting with the antibodies targeting TOP2 (Abcam, ab52934) for TOP2cc, and dsDNA (Abcam, ab27156) as the loading control. The quantification of TOP2cc was analyzed by measuring the density of the slot blot signal using image J software.

### 4.7. Immunoprecipitation

HEK293A cells expressing SFB tagged ZATT WT or mutants were lysed in a chilled 1× NETN buffer with proteinase inhibitors for 30 min on ice. Lysates were clarified by centrifugation at 13,000 rpm for 15 min. Then, 2% input samples were removed at this point and the lysates were incubated with streptavidin-conjugated beads (Fisher Scientific; Waltham, MA, USA) for 2 h at 4 °C on rotation. The beads were washed with a NETN buffer twice and boiled in an SDS sample buffer. Samples were loaded onto the SDS-PAGE and analyzed by western blotting with the indicated antibodies.

### 4.8. Statistical Analysis

Statistical significance was determined by Student *t*-tests (two tailed) or one-way ANOVA using GraphPad Prism8 software (GraphPad; Boston, MA, USA). Bars and error bars are presented as means ± S.D. with at least three times repeats.

## Data Availability

The data presented in this study are available upon request.

## Supplementary Materials

The following supporting information can be downloaded at: https://www.mdpi.com/article/10.3390/ijms24076545/s1.
