# Supplementary material for: Genome-Wide CRISPR Screens Reveal ZATT as a Synthetic Lethal Target of TOP2-Poison Etoposide That Can Act in a TDP2-Independent Pathway
Source: Int J Mol Sci. 2023 Mar 31;24(7):6545. doi: 10.3390/ijms24076545 (PMC10095316; doi:10.3390/ijms24076545)
Supplement: Supplementary file 1 [file ijms-24-06545-s001.zip › Supplementary Figure.pdf]

Figure S1

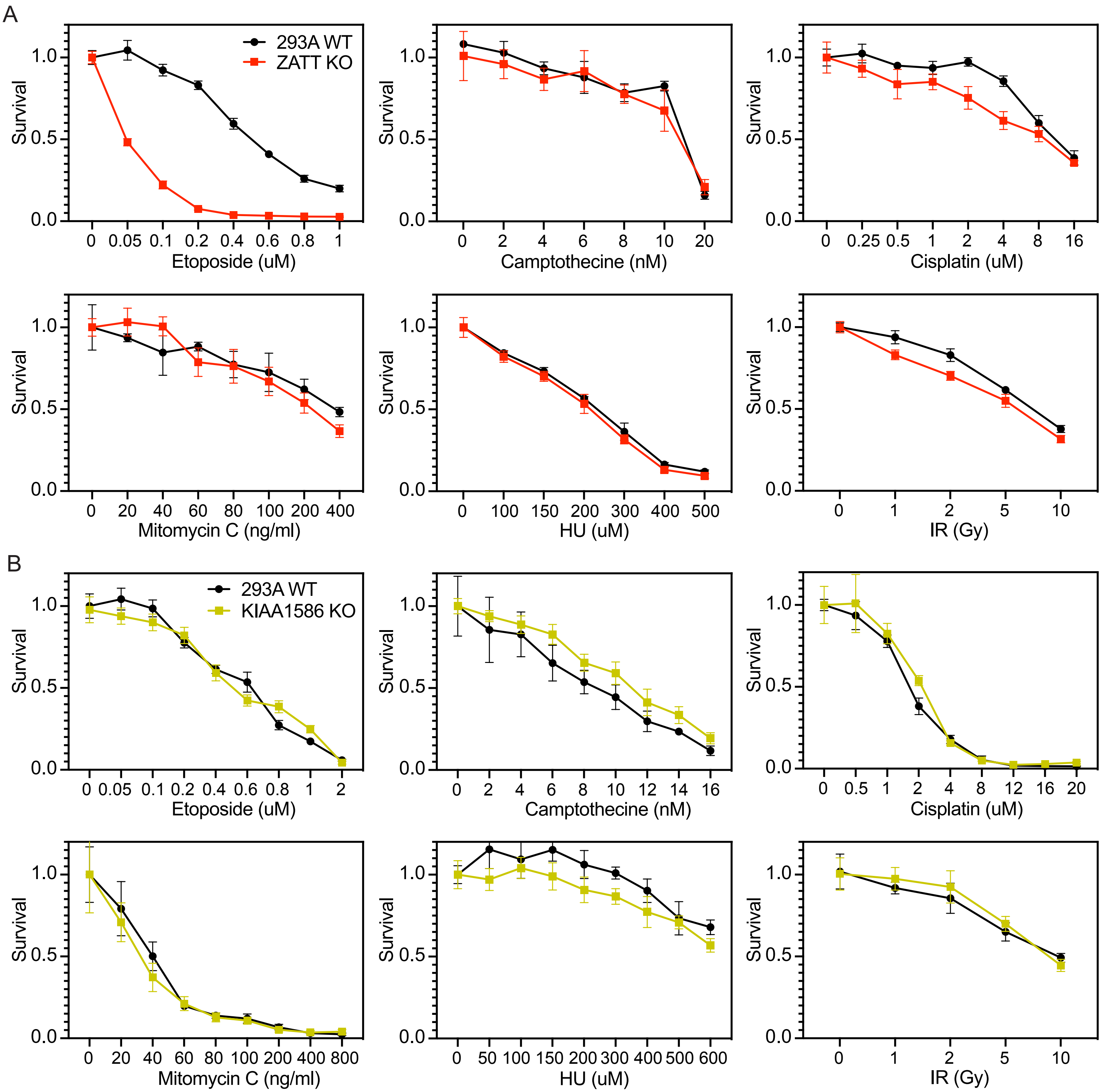

Figure S1. ZATT KO cells were sensitive to ETO, but not to other DNA damaging agents.
(A) Cell survival was measured using CellTiter-Glo assay in HEK293A WT and ZATT-KO cells treated with indicaed drugs for 3 days. Each experiment was repeated three independent times done in triplicates each time. Data were represented as mean ±SD. (B) KIAA1586 depletion did not change cell survival response to DNA damaging reagents. Cell survival was measured using CellTiter-Glo assay in HEK293A WT and KIAA1586 KO cells treated with indicated drugs for 3 days. Each experiment was repeated three independent times done in triplicates each time. Data were represented as mean ±SD.

Figure S2

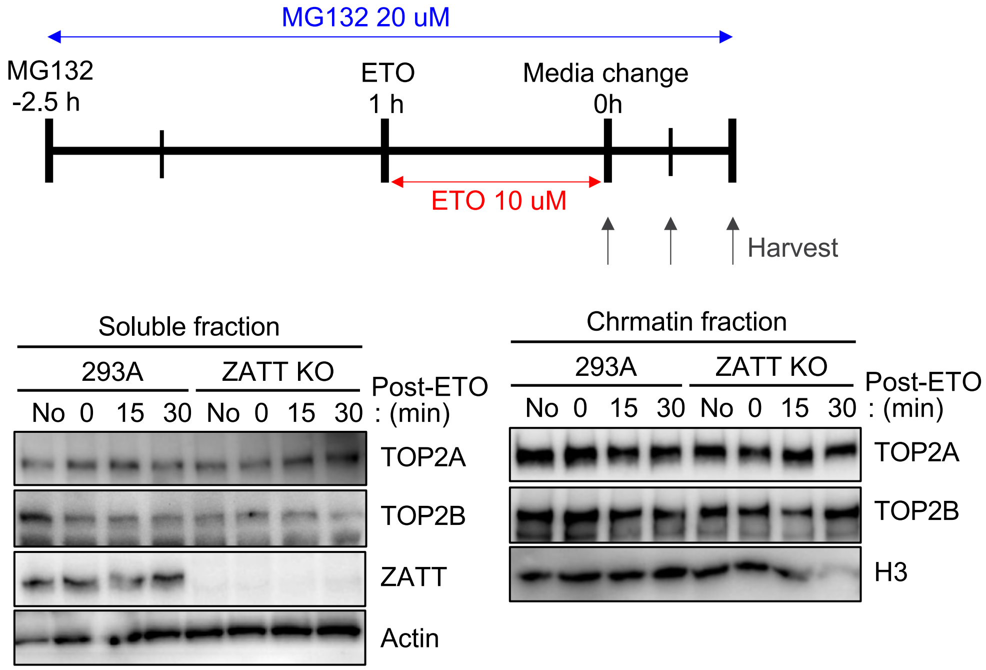

Figure S2. Proteolysis of TOP2α was suppressed by proteasome inhibitor MG132 both in WT and ZATT KO cells. Cells were treated with 20 uM MG132 and 10 μM ETO for indicated time. Soluble and chromatin fraction were prepared and subjected to Western blotting with the indicated antibodies.
